# Impacto de la formación en entrevista motivacional para médicos: diseño y evaluación de un Programa Formativo (MOTIVA)

**DOI:** 10.1016/j.aprim.2020.09.010

**Published:** 2021-03-18

**Authors:** Nieves Barragán-Brun, Remedios Martín-Álvarez, Josep M. Bosch-Fontcuberta, Manuel Campíñez-Navarro, Julia Bóveda-Fontan, Luis Ángel Pérula-de-Torres

**Affiliations:** a EAP Vallcarca, EBA Vallcarca, Barcelona, España; b EAP Encants, Institut Català de la Salut, Barcelona, España; c CS Colmeiro, SERGAS, Vigo, Pontevedra, España; d Instituto Maimónides de Investigación Biomédica-IMIBIC, Hospital Reina Sofía, Córdoba, España; e Miembro del Grupo Programa Comunicación y Salud de semFYC

**Keywords:** Entrevista motivacional, Formación médica continuada, Evaluación, Competencia professional, The motivational interview, Continuing medical education, Assessment training, Competence professional

## Abstract

**Objetivo:**

Desarrollar un programa de formación en Entrevista Motivacional (EM) para Médicos de Familia y evaluar el impacto.

**Diseño:**

Ensayo clínico multicéntrico a doble ciego y aleatorizado, con 2 ramas (experimental (GE) y control (GC)) de Médicos de Familia, con un seguimiento de 12 meses.

**Emplazamiento:**

32 Centros de Atención Primaria.

**Descripción de la muestra:**

54 médicos (GC = 28, GE = 26).

**Intervenciones:**

Programa de formación MOTIVA en EM con un curso presencial inicial (16 h), seguido de actividades en línea durante 12 meses y reuniones presenciales (entrevistas basadas en problemas con feedback de expertos).

**Medidas principales:**

las habilidades comunicativas en EM se evaluaron en base a videograbaciones (VG) con la escala EVEM 2.0, por parejas de revisores. Se analizaron 236 VG con pacientes estandarizados y 96 VG con pacientes reales.

**Resultados:**

Los resultados promedio en la escala EVEM (hasta 56 puntos) al inicio del estudio fueron GE = 21,27 (IC 95% 15,8-26,7) y GC = 20,23 (IC95% 16,4-23,9) sin diferencias entre ambos grupos (p = 0,79). Después del curso, la puntuación GE aumentó en 13,89 puntos (P <0,001), promedio 35,16 (IC 95% 29,8-40,6). Las VG de pacientes reales en GE durante el periodo de 12 meses mantiene sus habilidades en EM con un promedio de 36,9 puntos (IC 95% 30,3-43,6) versus GC 15,9 puntos (IC 95% 9,8-22,0). Una vez finalizado el Programa de Formación MOTIVA, el GE mantiene las habilidades adquiridas: GE promedio final = 37.6 (IC 95% 33.2-41.1) versus GC = 24,3 (IC95% 19,0-29,2) (p <0,001).

**Conclusiones:**

El Programa de Formación MOTIVA mejora significativamente las habilidades de entrevista motivacional, mejorando después de un curso presencial y actividades secuenciales de mantenimiento. La eficacia del programa ha sido probada en el tercer y cuarto escalón de la Pirámide de Miller.

## Abstract (en)

**Aim:**

To develop a training program in Motivational Interviewing for Family Physicians and assess the impact.

**Study design:**

Multicenter, double blind and randomized clinical essay, with 2 arms, Experimental (EG) and Control (CG) of Family Physicians with a follow up of 12 months.

**Location:**

32 Primary Healthcare Centers.

**Sample description:**

54 physicians (CG = 28, EG = 26).

**Interventions:**

Training Program MOTIVA in ME with an initial presential course (16 h), followed by online activities during 12months, and presential meetings (Problem BasedInterviewing with expert feedback).

**Main measurements:**

Communicative skills in MI were assessed based on video-recordings (VR) with the EVEM 2.0 scale by peer reviewers. 236 VR with standardized patients and 96 VR with real patients.

**Results:**

Average results in EVEM scale (up to 56 points) at the beginning of the study were EG = 21.27 (CI 95% 15.8-26.7) and CG = 20.23 (CI95% 16.4-23.9) with no differences between both groups *(P*=.79). After the training, EG punctuation increased by 13.89 points *(P*<.001), average 35.16 (CI 95% 29.8-40.6). Real patients’ VR in EG over a 12 month period keep their MI skills with an average of 36.9 points (CI 95% 30.3-43.6) versus CG 15.9 points (CI 95% 9.8-22.0). Once ended the MOTIVA Training Program, the EG maintains the acquired skills: final average EG = 37.6 (CI 95% 33.2-41.1) versus CG = 24.3 (CI95% 19.0-29.2) *(P*<.001).

**Conclusions:**

The MOTIVA Training Program improves Motivational Interviewing skills, significatively improving after a presential course and sequential keep-alive activities. The effectiveness of the Program has been proven in the Third and Fourth steps of Miller's Pyramid

## Introducción

Los profesionales sanitarios orientan a sus pacientes sobre hábitos y estilos de vida haciendo uso de sus habilidades en comunicación. Cabe preguntarse si la formación en entrevista clínica podría mejorar esas habilidades y qué tipo de actividades formativas serían las más efectivas para mejorar esta competencia profesional esencial. Las revisiones de la literatura sobre estudios de efectividad en *Educación Médica Continuada,* valoran hasta 8 tipos de intervenciones formativas^1^ (guías, conferencias y talleres, encuentros con formadores, opinión de colegas, opinión de pacientes, audit/feedback, recordatorios de actividades e intervenciones multifaceta). Destacan 3 aspectos fundamentales para el aprendizaje: el profesional debe sentir la necesidad de aprender^2^, ^3^, el formato debe ofrecer la posibilidad de *practicar habilidades*^2^, ^4^ y el *diseño* de las actividades formativas debe ser *multifacético* y secuenciado^2^, ^5^. Los estudios concluyen que las sesiones didácticas aisladas no tienen impacto en la práctica habitual.

La entrevista motivacional (EM) desarrollada por W. Miller y S. Rollnick (1983) es uno de los modelos de comunicación clínicos más estudiados para favorecer cambios de conducta^7^. No hemos encontrado en el entorno de Atención Primaria (AP) estudios con programas formativos multifacéticos en EM ni que utilicen las videograbaciones (VG) en la consulta para su evaluación.

El presente estudio plantea el diseño del *Programa Formativo MOTIVA (PF MOTIVA) en EM en el entorno de la AP*, con el objetivo de responder 2 preguntas clave: *¿la formación puede* *mejorar el perfil comunicativo de los médicos para motivar a los pacientes?, y ¿qué tipo de acciones formativas son más efectivas?* El análisis se centró en el impacto del programa en la práctica clínica de médicos de Familia (MF).

## Metodología

Diseño del estudio: ensayo clínico multicéntrico, abierto, controlado, a doble ciego y aleatorizado por clúster, con 2 brazos paralelos formados por MF con el objetivo de estudiar el impacto de un programa formativo en EM.

**Figura 1:**
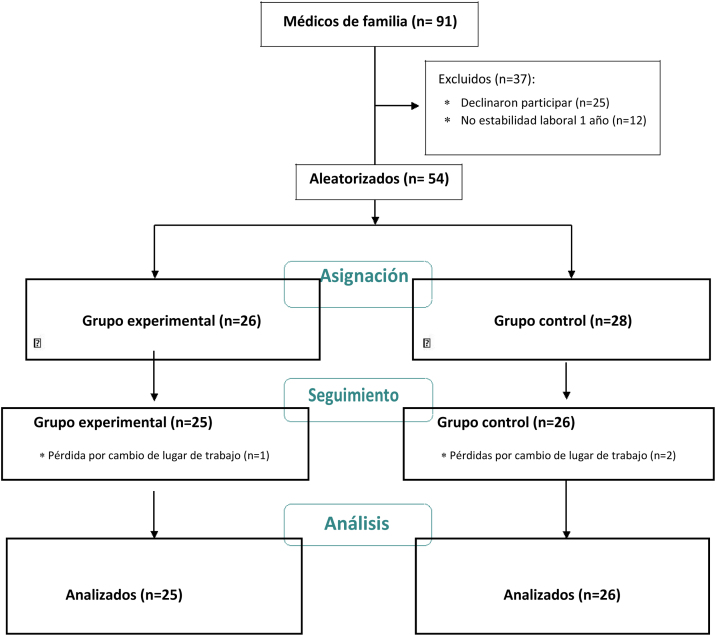

Participantes: fueron seleccionados por muestro de conveniencia *54 MF* en Barcelona, Córdoba, Vigo y algunos pueblos cercanos, para participar en un estudio sobre manejo de pacientes con dislipidemia (Dislip-EM^8^). Inicialmente, recibieron formación en las guías de práctica clínica y, posteriormente, se distribuyeron por aleatorización simple (Epidat 3. [fig. 1]. Todos, grupo control (GC) y grupo experimental (GE), incluyeron a pacientes que fueron atendidos con *el estilo habitual de entrevista clínica y consejo médico (GC)* o con *el modelo de EM (GE).* Los pacientes podían ser tributarios o no de tratamiento farmacológico según las guías. Se realizó un seguimiento de 12 meses. *El PF MOTIVA* fue la intervención realizada al GE del Dislip-EM.

Criterio de inclusión: previsión de trabajo del MF en el centro de salud durante 12 meses.

**Figura 2:**
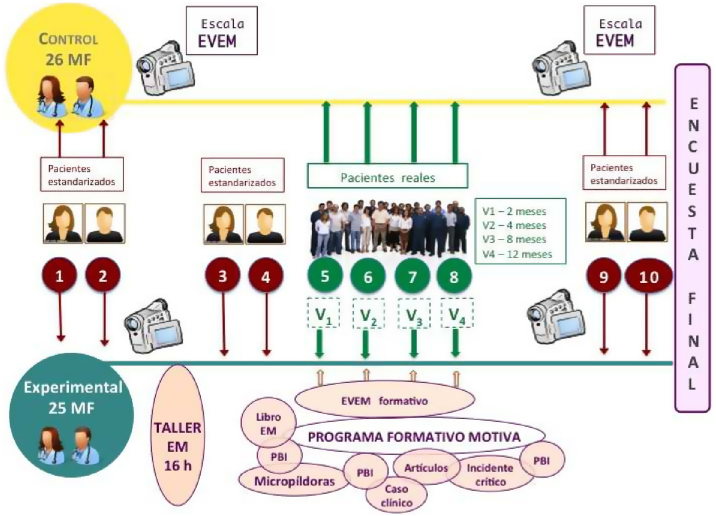
Esquema gráfico de PF MOTIVA.

Intervención: PF MOTIVA. Dirigido por expertos en EM y coordinado desde una unidad docente de MF. Incluyó las actividades: *curso de EM* presencial de 16 h siguiendo las 8 etapas de aprendizaje de Miller y Moyers^9^, y utilizando metodología interactiva con role-play. Se realizó consecutivamente en las 3 ciudades citadas antes de la captación de pacientes. *Lectura del libro* La entrevista motivacional^10^. *Lectura de 5 artículos* sobre EM. *Ejercicio escrito de reflexión sobre 2 casos clínicos enviados.* Recepción de *micropíldoras formativas* por email y WhatsApp cada 15 días. Redacción de un *incidente crítico* sobre EM acaecido en su consulta y feedback posterior. *Sesiones de problem based interviewing (PBI)*: técnica de análisis de VG mediante el feedback grupal y la autorreflexión. Se ofrecieron 3 sesiones en la que cada MF presentó una de sus VG (fig. 2).

Cada MF se *videograbó* con uno de sus pacientes (el 3.° incluido de manera aleatoria) en todas las visitas de seguimiento (12 meses). Se videograbaron 2 entrevistas con pacientes estandarizados (PE) antes del PF MOTIVA, tras el curso y al final del PF MOTIVA. Los casos clínicos «simulados» se diseñaron con metodología ECOE^11^ centrados en hábitos de vida y factores de riesgo cardiovascular.

Variables de estudio y medidas de evaluación. Se recogieron los datos demográficos de los MF (edad, sexo), los años trabajados en AP y la formación en comunicación, específicamente en EM (tabla 1).

**Tabla 1:** Características sociodemográficas de los médicos participantes

variables	Grupo experimental n = 25	Grupo control n = 26	Valor de p
*Edad, media ± DE*	45,5 ± 8,3	47,6 ± 7,5	0,36
*Sexo, n (%)*			0,55
Mujeres	11 (44)	12 (46,2)	
Hombres	15 (56)	14 (53,8)	
*Años de práctica asistencial (sin residencia), n (%)*			0,46
2-5	2 (8)	2 (7,7)	
11-15	4 (16)	2 (7,7)	
6-10	3 (12)	3 (11,5)	
16-20	4 (16)	7 (26,9)	
21-25	5 (20)	2 (7,7)	
> 25	4 (16)	9 (34,6)	
NS/NC	3 (12)	1 (3,8)	
*Formación en entrevista clínica, n (%)*			0,31
No	3 (12)	1 (3,8)	
Sí	22 (88)	25 (96,2)	
*Formato de formación recibida en entrevista clínica, n (%)*			0,43
Presencial	20 (80)	21 (80,8)	
Ambas (online + presencial)	2 (8)	4 (15,4)	
NS/NC	3 (12)	1 (3,8)	
*Horas de formación presencial en entrevista clínica, n (%)*			0,77
0	3 (12)	1 (3,8)	
1-4	2 (8)	1 (3,8)	
5-8	1 (4)	1 (3,8)	
9-12	1 (4)	1 (3,8)	
13-16	1 (4)	4 (15,4)	
17-20	8 (32)	11 (42,3)	
> 20	5 (20)	3 (11,5)	
NS/NC	4 (16)	4 (15,4)	
*Formación específica en entrevista motivacional, n (%)*			0,89
Sí	12 (48)	13 (50)	
No	13 (52)	13 (50)	

La *evaluación del PF MOTIVA* fue mediante el análisis de las VG utilizando la *escala EVEM 2.0,* que valora la calidad o el grado de adherencia a la EM^12^. Se analizó el comportamiento de los MF en consultas simuladas con PE, que corresponde al nivel de competencia clínica del 3.^er^ *escalón de la pirámide de Miller*^13^, ^14^ *(«Demuestra cómo hacerlo»)* y en consultas con sus pacientes reales (PR), en condiciones clínicas habituales, el *4.° escalón de Miller («Lo hace en situaciones habituales»).*

Los pacientes firmaron el consentimiento de cesión de imagen y utilización en el entorno docente. Las VG fueron anonimizadas para la aleatorización y revisión por pares de expertos. La videoteca estuvo en *vimeo.com,* con su correspondiente clave de acceso para cada VG. Los evaluadores recibieron un listado de enlaces a vídeos y claves de acceso. Las puntuaciones de la escala EVEM se introdujeron en un formato online. La base de datos recogía para cada MF: evaluación EVEM de sus vídeos, actividades formativas realizadas, datos demográficos y encuesta final de evaluación.

Se diseñó *un indicador sintético (IS) de adherencia al PF MOTIVA* para dar valor a las 14 *herramientas docentes en comunicación,* y que valora la potencia formativa de cada una de ellas en el impacto sobre el aprendizaje en EM. Se creo un comité de expertos con 17 profesionales docentes en comunicación clínica seleccionados por su trayectoria y prestigio dentro del grupo comunicación y salud. Un experto matemático elaboró una fórmula que dio un valor numérico para la ponderación de cada actividad.

Encuesta de satisfacción. Al final del estudio se realizó una encuesta online de valoración del proyecto que abordó aspectos logísticos y de factibilidad del estudio, así como la percepción de utilidad para el aprendizaje de cada una de las actividades o herramientas docentes.

Medidas de evaluación. Es un estudio por intención de tratar, siendo la unidad de estudio el MF. Se realizó el análisis comparativo basal de ambos grupos empleando la prueba de la t de Student y de la ji al cuadrado. El análisis de adquisición de habilidades se realizó con el test ANOVA para medias pareadas en diferentes momentos: tras el taller de EM, en 4 visitas anuales y al final.

Se consideraron estadísticamente significativos los valores p < 0,05. Se calcularon los intervalos de confianza del 95% (IC del 95%). El análisis estadístico se realizó con el programa SPSS v23 de IBM.

## Resultados

Participaron 54 médicos, distribuidos aleatoriamente en GE = 26 y GC = 28. Hubo una pérdida en GE y 2 en GC. *La adherencia al PF MOTIVA* fue: taller presencial 100%, feedback experto al vídeo con PE tras taller 100%, PBI 62%, micropíldoras 64%, lectura (libro y artículo en EM de Miller y Rollnick 33%), lectura artículo «Fumar es un placer» 24%, informe de autorreflexión 56%, reflexión caso clínico 52%, incidente crítico 32%, VG y autoevaluación entrevista 32%.

La tabla 2 muestra la descripción de variables. Se analizaron 332 (VG): GC = 140, GE = 192. VG con PE: preintervención GC = 48 y GE = 46, postaller GE = 48 VG, al finalizar GC = 47, GE = 49. VG con PR: Durante los 12 meses GC n = 45, GE n = 49.

**Tabla 2:** Videograbaciones realizadas en cada fase del PF MOTIVA

Grupo	Fase 1. Nivel EM basal	Fase 2. Nivel EM postaller	Fase 3. Atención Primaria: PR	Fase 4. Nivel EM final	VG total
Control (n = 26)	48	0	45	47	140
Experimental (n = 25)	46	48	49	49	192
Total	**94**	**48**	**94**	**96**	332

Las medias para la escala EVEM (máximo 56 puntos) antes del estudio fueron GE = 21,27 (IC del 95%, 15,8-26,7), un 38% del total (IC del 95%, 28-47%) frente a GC = 20,23 (IC del 95%, 16,4-23,9), un 36% del total (IC del 95%, 29-42%), sin diferencias entre ambos grupos (p = 0,79). Eficacia en el tercer escalón de la pirámide de Miller: Tras el curso, la media con EVEM fue de 35,16 (IC del 95%, 29,8-40,6), un 63% del total (IC del 95%, 53,2-72,5), lo que supone un aumento de 13,89 puntos en la media de la escala (IC del 95%, 6,9-19,5) (p < 0,001). Al final del PF MOTIVA, la media GE fue 37,6 (IC del 95%, 33,2-41,1), un 68% del total (IC del 95%, 59,3-75,1) frente a la media del GC que fue de 24,3 (IC del 95%, 19,-29,2), un 43% del total (IC del 95%, 35,1-51,9), con una diferencia significativa (p < 0,001).

Eficacia en el cuarto escalón de la pirámide de Miller: la media de GE fue de 36,9 (IC del 95%, 30,3-43,6), un 64% del total (IC del 95%, 54,5-73,4) frente al GC, que fue de 15,9 (IC del 95%, 9,8-22), y un total de 28,2% (IC del 95%, 16,9-39,5). Estas diferencias fueron estadísticamente significativas (p < 0,001), con una diferencia de medias de 21 puntos (19,2-22,8) (tabla 3).

**Tabla 3:** Valoración de las videograbaciones de médicos de familia con pacientes estandarizados y encuentros reales, utilizando la escala EVEM 2.0

Variables	Grupo experimental n = 25 (ptos ± DE), %	Grupo control n = 26 (ptos ± DE), %	Diferencia de medias (IC del 95%)	Diferencia de medias (%)	Valor p
*Tercer escalón pirámide Miller (videograbaciones con pacientes estandarizados)*					
EVEM inicial, media ± DE (%)	21,27 ± 12,34 (38%)	20,23 ± 8,54 (36%)	1,04 (0,6 a 2,8)	2%	0,79
EVEM postaller, media ± DE (%)	35,16 ± 12,73 (63%)	–	–	–	
EVEM final, media ± DE (%)	37,64 ± 10,40 (68%)	24,37 ± 10,98 (43%)	13,27 (11,9 a 14,2)	25%	0,001
Diferencia de medias pareadas inicial_final (12 meses) (IC del 95%)	16,37 (14,4-17,4)	4,14 (2,6-5,3)	–	–	

*Cuarto escalón pirámide Miller (videograbaciones en encuentros clínicos con pacientes reales)*					
EVEM paciente real 2-4 meses, media ± DE (%)	35,43 ± 12,6 (62%)	15,8 ± 11,5 (31%)	19,6 (18,53 a 21)	31%	0,001
EVEM paciente real 8- 12 meses, media ± DE (%)	35,8 ± 11,4 (66%)	16,7 ± 12,6 (31%)	19,1 (17,9 a 20,3)	35%	0,001
EVEM paciente real global, media ± DE (%)	36,9 ± 12,9 (64%)	15,9 ± 11,1 (28%)	21 (19,2-22,8)	36%	0,001

**Figura 3:**
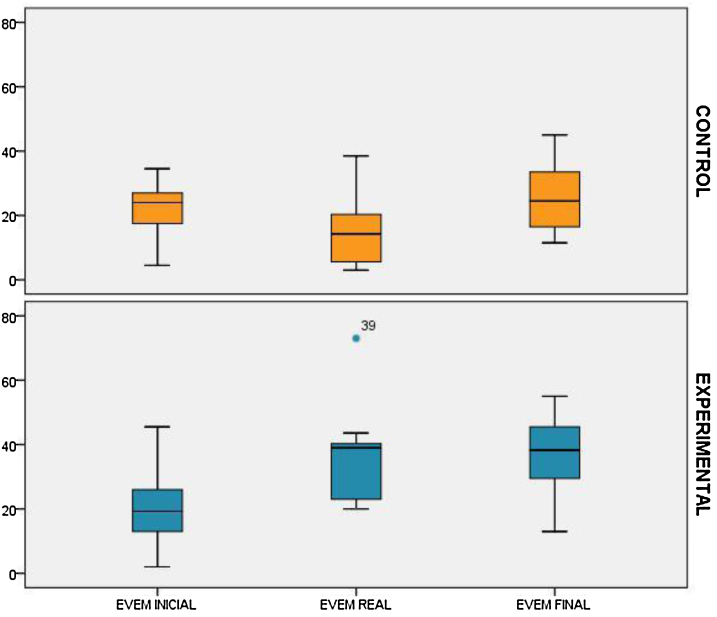
Comparación de la escala EVEM con la implantación del Programa MOTIVA.

La figura 3 muestra cómo los cambios en el GE se mantienen en el tiempo. *El valor del IS* fue de mayor a menor: PBI 3,44, feedback de experto 3,33, taller interactivo presencial 2,91, incidente crítico 2,81, autoevaluación de la VG 2,55, informe de autorreflexión 2,41, comentario escrito sobre caso clínico de comunicación 2,2, libro de entrevista clínica 1,89, artículo sobre comunicación 1,42, recibir micropíldoras formativas 1,39 y clase magistral 0,96.

El taller de EM es la actividad con mayor impacto junto a la realización de sesiones PBI (p = 0,024). *El perfil competencial* de los participantes al inicio del programa no se relacionó con la adherencia al PF MOTIVA (p = 0,82), siendo la escala EVEM al final del PF MOTIVA la que presenta una relación positiva con la adherencia (p = 0,017).

Respecto a la formación previa, el 88% del GE había realizado el curso básico de entrevista clínica durante la residencia MIR y el 96% del GC. Respecto a la EM, el 88% había realizado formación presencial en el GE y 96,2 en el GC. Los resultados de la encuesta de satisfacción señalan que el 100% de los participantes tuvieron una clara percepción de mejora en la relación con sus pacientes y en la incorporación de nuevas habilidades de EM. Las 3 herramientas más valoradas para aprender fueron: taller EM (88%), PBI (64%) y feedback personalizado (60%). El 100% recomendaría este programa a sus compañeros.

## Discusión

Existen pocos estudios comparables con el PF MOTIVA y ninguno en nuestro país. El tamaño muestral es similar o mayor al de otros estudios de formación en EM en AP^15^, ^16^, ^17^. En la mayoría de ellos utilizan audiograbación y solo algunos VG, aunque con una muestra menor que esta^18^, ^19^, ^20^, ^21^, ^22^. El estudio EMMEE (Miller et al.^23^, 2004) tiene un diseño similar con un taller de 2 días para profesionales del entorno de abuso de sustancias, con 5 brazos que incluye, entre otros, feedback escrito a las audiograbaciones de PR, otro coaching telefónico o ambos. Las conclusiones muestran cómo decae el aprendizaje 4 meses tras el taller. Sin embargo, el feedback y el coaching mantienen dicho aprendizaje.

Durante los 12 meses de desarrollo del PF MOTIVA, las habilidades en EM valoradas mediante la escala EVEM muestran una estabilidad en el tiempo, sin cambios intragrupos (fig. 3).

En el entorno de AP, la escala EVEM representa la mejor opción para evaluar el aprendizaje del estilo motivacional porque tiene validez aparente, de consenso, de contenido, ha demostrado una elevada consistencia interna, excelente reproductibilidad y validez convergente con la escala BECCI^24^. La misma escala permite evaluar PE y PR. Así, asumimos que con los PE se evalúa el tercer escalón de Miller y con los PR evaluamos el cuarto.

En un programa formativo largo y con actividades muy variadas, inmerso en el contexto laboral, preocupó *la factibilidad de su desarrollo*. La viabilidad en el contexto de la AP se fue demostrando a medida que avanzaba el programa, consiguiéndose la participación de todos los MF sin diferencias por dispersión geográfica. La encuesta de valoración final refleja esta factibilidad del programa. Las nuevas tecnologías han sido un elemento clave en la facilitación del desarrollo de las diferentes actividades^25^, destacando el trabajo del equipo coordinador que se reunía periódicamente online (Second Life, Skype).

El diseño multicéntrico simultáneo del programa cuidó diversos aspectos formativos: contenidos teóricos de EM, autorreflexión sobre práctica clínica, feedback docente, entrenamiento de habilidades comunicativas, participación activa. Se intercaló el trabajo presencial, online, individual y de equipo.

El PF MOTIVA ha integrado las actividades con más evidencia en el impacto del aprendizaje en la bibliografía internacional. No ha incluido charlas magistrales con menos impacto en habilidades, optando por herramientas más interactivas como talleres^26^ con role-play^27^, feedback docente^28^ con metodología PBI^29^, utilización de PE^13^, incidentes críticos, estudio caso clínico y lectura del libro de referencia en EM^12^, y de otros artículos de interés. Se han utilizado recordatorios de conceptos relevantes con un listado imantado adherido al ordenador que recordaba las tareas fundamentales de la EM y micropíldoras formativas por WhatsApp y email.

El curso de la EM ha sido la actividad más relevante y una de las mejor valoradas^9^. El ambiente participativo potenció la aportación de experiencias clínicas y el desarrollo de habilidades comunicativas. Cada MF analizó y trabajó el proceso de «desaprender», cuando era necesario, con el objetivo de asimilar e integrar el estilo motivacional en su práctica clínica. No es posible separar el impacto aislado del taller y de los PBI debido a la diferente adherencia al programa de los participantes, por la participación irregular a lo largo del año en función de situaciones personales. Para aislar este impacto debería plantearse un estudio donde estas 2 actividades fueran una rama diferenciada de investigación. Algunos estudios que incluyen solamente un taller también encuentran un incremento del perfil motivacional, pero no realizan seguimiento a largo plazo^23^.

Las sesiones de visualización grupal de VG con metodología PBI^29^ se incluyeron en el PF MOTIVA por la experiencia del grupo coordinador (Programa Comunicación y Salud de SemFYC). Son sesiones donde se comparten el conocimiento y la experiencia de la comunicación médico-paciente con la percepción unánime de generar un gran aprendizaje: el análisis de VG de la propia consulta junto con el feedback de los compañeros y un experto.

Aparte del taller de EM inicial, la actividad que mayor impacto ha demostrado es la participación en las sesiones de PBI (p = 0,024) Las 3 actividades más valoradas por los expertos en la construcción del IS (taller teórico-práctico, feedback docente y PBI) coinciden con la percepción de los participantes en la utilidad para aprender habilidades de comunicación.

La evaluación sumativa con la escala EVEM determina si el participante ha alcanzado los objetivos específicos de adquisición de habilidades en EM. La evaluación inicial de competencias (baseline) está presente también en muchos estudios de intervención en la formación en EM^15^, ^16^, ^23^, ^30^. Una vez finalizado el curso de EM, se objetivó la adquisición de habilidades en EM con PE de manera significativa. Estos resultados son congruentes con estudios similares^15^.

No encontramos estudios que puedan corroborar la estabilidad en el aprendizaje. El estudio de Baer (2004) mostró una pérdida de habilidades 2 meses después de realizado el curso. Las tareas posteriores, secuenciadas y con feedback probablemente han mantenido las habilidades, permitiendo incorporarlas como hábito en la entrevista clínica. El esfuerzo de los MF por grabarse con sus pacientes y dejarse evaluar por expertos demuestra un interés genuino por aprender. Tal vez el sesgo «del voluntario» influya en la gran adherencia que ha presentado el PF MOTIVA.

La valoración de los encuentros reales demostró diferencias entre ambos grupos y una tendencia mantenida durante 12 meses (GC y GE). El análisis de las VG de los PR del GE objetiva que las habilidades adquiridas durante el taller de EM se llevan a la práctica clínica diaria, mientras el GC sigue utilizando sus habilidades habituales.

Los resultados de la encuesta de satisfacción señalan que todos los participantes tuvieron una clara percepción de mejora en la relación con sus pacientes y en la incorporación de nuevas habilidades de EM, lo que nos estimula en esta línea docente. La eficiencia no se evaluó en este proyecto, sin embargo, ya que no precisó materiales específicos, el coste se acotó al curso formativo y a las sesiones de feedback docente. Los PE no forman parte del diseño del programa, sino de su evaluación, por lo que no se contabilizan como coste de formación. En contraposición a los costes, el PF MOTIVA ofrece a los médicos una herramienta muy útil para problemas de salud prevalentes en AP relacionados sobre todo con hábitos de vida no saludables. Permite alternativas al tratamiento farmacológico y estimula el autocuidado de los pacientes, factores que aumentan la eficiencia.

Para concluir, podemos afirmar que el PF MOTIVA diseñado y evaluado en este proyecto tiene gran aplicabilidad en el entorno profesional de la AP, ha demostrado su factibilidad, los MF han expresado su satisfacción al concluirlo y ha mostrado un claro impacto en la aplicación del modelo comunicativo de la EM.

## Financiación

Este trabajo recibió una Beca Isabel Fernández 2012 de la semFYC.

## Conflicto de intereses

Los autores declaran no tener ningún conflicto de interés relacionado con el contenido del manuscrito.
